# Knowledge mobilization between the food industry and public health nutrition scientists: findings from a case study

**DOI:** 10.1186/s40795-024-00889-z

**Published:** 2024-06-06

**Authors:** Marie Le Bouthillier, Sophie Veilleux, Jeanne Loignon, Mylène Turcotte, Laurélie Trudel, Véronique Provencher

**Affiliations:** 1 Centre NUTRISS—Nutrition, Santé et Société, Institut sur la Nutrition et les Aliments Fonctionnels (INAF), Université Laval, 2440, Hochelaga Boulevard, Pavillon des services, office 2729-L Qc, Quebec, QC G1V 0A6 Canada; 2 École de Nutrition, Université Laval, 2440, Hochelaga Boulevard, Pavillon des services, office 2729-L Qc, Quebec, QC G1V 0A6 Canada; 3 Faculté des sciences de l’administration, Université Laval, Quebec, QC G1V 0A6 Canada; 4 Institut sur la Nutrition et les Aliments Fonctionnels (INAF), Université Laval, 2440, Hochelaga Boulevard, Pavillon des services, office 2729-L Qc, Quebec, QC G1V 0A6 Canada

**Keywords:** Knowledge mobilization, Knowledge transfer, Food industry, Public health researchers, Nutrition knowledge

## Abstract

**Background:**

Improving the nutritional quality of the food supply increases access to nutritious foods, which improves dietary habits and population health. Yet, knowledge mobilization initiatives between public health nutrition researchers and food industries are often not adequately considered and understood. This study explored what elements related to this specific context need to be recognized so that researchers can better mobilize nutrition science knowledge with the food industry to promote the nutritional improvement of food products.

**Method:**

A case study qualitative approach was selected to answer the research question, using semi-structured interviews as the data collection technique. Québec baking industry actors were shown a mock-up of an online mobilization platform sharing the results of the Food Quality Observatory that describes the nutritional quality of breads offered in Québec, Canada. They were asked to think aloud as they explored the web platform and were interviewed. Two coders analyzed the data using an inductive approach and thematic content analysis, starting with individual open coding, and then put forward their analyses and drafted the final themes.

**Results:**

The final data consisted of 10 semi-structured interviews conducted between October 2019 and August 2020. Four main themes were identified: the industry’s context, the knowledge mobilization initiative, the product-related matters stemming from the information shared and the motivation within the industry. Within each theme, sub-themes were highlighted and related to the industries’ motivation to improve their products’ nutritional quality. This study also specified key considerations for changes to the sodium and fiber content in bread.

**Conclusion:**

Other steps beyond using simple language and a website format could be taken to better mobilize scientific knowledge with food industries, such as providing more consumer information, using an integrated knowledge mobilization approach that includes a consideration of ethics, working with communication professionals, collaborating with food science experts, and providing resources to act on shared information. Legislation such as the front-of-pack regulations could accelerate the pace of collaboration between researchers and industry. Overall, establishing a prior relationship with industries could help gain a better understanding of the themes highlighted in this study. Future research could build on this case study to provide more insights and solidify these findings.

**Classification codes:**

Public Health, Public Private, Policy Making, Research Institutions, Use of Knowledge.

**Supplementary Information:**

The online version contains supplementary material available at 10.1186/s40795-024-00889-z.

## Introduction

Improving the nutritional quality of the food supply, meaning improving the nutrient profile of food products, can enhance public health through better access to nutritious foods for consumers [1]. Public health researchers are involved in these improvements via the emerging role of universities to contribute to society, through activities that apply their knowledge toward innovation in businesses or organizations [2]. However, a gap remains between public health nutrition researchers’ knowledge of the potential areas of nutritional improvement in foods, and their application by food businesses. Frequent assessment of the nutritional quality of the food supply can attest to this, as there is a disparity between what is currently offered and what scientists have determined to be a nutritious food supply [3, 4]. Although public health researchers are not the only scientists who could share their knowledge for the benefit of the nutritional quality of foods, to our knowledge they have not yet been directly studied in the literature, despite their primary role in generating studies on public health in relation to food reformulation [1, 5].

To address this disparity, public health nutrition researchers should engage in more collaborative activities with food industries, such as knowledge mobilization (KM) initiatives. KM is a broad term that includes many activities such as sharing, synthesizing, exchanging, and co-producing knowledge between producers and users [6]. In a scientific context, the goal is for research and evidence to inform decisions and an understanding of public policy, professional practice, and other applications to transform research into action [7]. An example of a positive KM outcome between food industries and nutrition researchers is that scientific knowledge in the field of nutrition leads to healthier products being produced by the food industry [8].

The present study investigated the case of a KM initiative between public health researchers in nutrition and the baking industry after the provision of scientific information; however, the findings can help inform situations with other types of food businesses or other public health organizations and at different stages of KM. The aim of this study is to explore elements related to a KM initiative between public health researchers and the food industry, including the industry’s context prior to KM, the KM initiative itself including the knowledge shared, the product-related issues involved in improving the nutritional value of products, and the industry’s motivation. Highlighting key elements to guide knowledge mobilization approaches will help researchers and research organizations better use their findings for the benefit of public health. Food businesses could also benefit from this research, as healthy and improved products are increasingly in demand among consumers [9]. It can also potentially improve policy and legislation by highlighting sensitive issues for industries regarding the nutritional improvement of food products.

## Literature review

Generally speaking, the best practices to specifically engage in KM involves an initial attempt to understand the needs of information users [6]. Moreover, regardless of the type of industry, there are several barriers to mobilizing knowledge generated by scientists, including the knowledge user’s capacity to absorb scientific knowledge, the ambiguity of information, differing goals, and trust issues between organizations [10–12]. Whether these aspects of KM apply to the specific context of food industries and public health researchers is unclear due to food sector characteristics.

KM initiatives in food industries may have additional or different specifications than those made with other types of businesses, as this sector has certain particularities. Food innovations include technological, social, and cultural dimensions, and the food industry is a low-tech sector that is primarily motivated by improving its products in response to consumer demands [13–16]. In addition, companies produce products with short shelf lives; therefore, selling these foods quickly is paramount [17]. Finally, the food industry is highly dependent on ingredient suppliers [18, 19].

In terms of the type of knowledge shared in KM, most studies to date on food industry innovation processes involve knowledge coming from either food science or food engineering, but not from nutrition sciences [20, 21]. Food products have seen major improvements in packaging or in the biotechnologies used to produce them, while incremental improvements in the nutrient profile of staple foods have yet to be realized [3, 4, 21]. As such, it has been shown that dietitians and other nutrition professionals can improve their knowledge translation skills [22]. One study conducted in the context of a collaboration between five nutrition researchers and five food companies revealed positive attitudes toward collaboration, but special attention must be paid to common goals, trust, prejudice, and collaboration agreements [23]. Outside this study, little is known about the key elements of a KM initiative to consider, such as the context, barriers, and attitudes to be aware of when sharing nutrition science with the food industry, rather than knowledge about innovative technologies or ingredients.

Ethical issues are often a concern for nutrition researchers in collaborating with the food industry, especially regarding scientific independence and rigor [24–26]. Many guidelines are available to address these concerns and to guide actions and partnerships with the food sector. For example, the American Society for Nutrition suggests a set of twelve principles to ensure integrity in the pursuit of research with food business research [27]. Overall, it is recommended to work toward better collaboration between public health organizations and industries to create a healthy and sustainable food system and to address and overcome those ethical concerns, rather than not collaborate [8, 28, 29]. As such, much research has focused on how industries can influence public health and nutrition policy [30], but little research has been conducted on how scientists can positively influence industries in return, by mobilizing their knowledge effectively with positive public health outcomes.

## Materials and methods

A qualitative approach was selected for this study, given the exploratory objective of investigating key elements that public health nutrition researchers should consider to better mobilize their knowledge with the food industry and encourage nutritional improvement of food products [31–33]. The grounded theory approach guided the philosophical research perspective, while the COREQ guidelines helped with the reporting of the study [34, 35]. This study was defined as a case study, given the limitations of what has been studied and that this study represents a preliminary analysis of this delimited context, which is intended to foster new hypotheses and research questions [36]. The data collection technique selected was individual semi-structured interviews along with the think-aloud method, which allows for rich insights and the possibility of follow-up questions by interviewers [37, 38]. The *Comité d’éthique de la recherche avec les êtres humains de l’Université Laval* (#2019 –225 A-1/11-05-2020) approved this study. All methods were carried out in accordance with the applicable guidelines and regulations. Informed consent was obtained from the study participants.

The case study was a KM approach between the Québec baking industry, represented by the *Conseil de la Boulangerie du Québec* (CBQ), and nutrition researchers from the Food Quality Observatory (Observatory). The CBQ is a non-profit organization that includes business members from the baking and pastry sector of the Québec food-processing industry. Their mission is to support the industry’s development and represent it at various levels of government. The Observatory’s mission is to monitor the food supply to improve food quality and accessibility [39]. Among its activities, which are funded by the government of Québec, the Observatory conducts studies on the nutritional quality of the different food categories most consumed by Quebecers (https://offrealimentaire.ca/en).

In 2019, the Observatory team presented the results of a study on the nutritional quality of sliced bread at a general meeting attended by a member of the *Conseil de la Transformation Alimentaire du Québec* (CTAQ), of which the CBQ is a subcommittee. The members then asked the researchers to present the results to the CBQ members directly. This request was made because these results could benefit this sector, as a potential front-of-pack (FOP) regulation for high-sodium products could be implemented by Health Canada (Gouvernement du Canada, 2018, Gouvernement du Canada, 2022). This would impact sliced bread packages since 27% of products studied were above the 15% daily value for sodium, meaning that they would have to potentially bear an FOP indication [3]. Thus, the Observatory team verbally presented the study results to some CBQ members, who suggested that the information could be simplified. The researchers decided to create the current research project, where workers from CBQ member industries would be interviewed by the research team, to better address and understand this issue.

Before recruiting these participants, the results of the Observatory’s study were presented on an online platform. The platform was created by the research team, with the aim of simplifying the data presented and inviting participants to become involved in improving the nutritional quality of the products offered by the food business for which they work. The platform was written in French, as all participants spoke French as their first language, as did the research team. The platform consisted of four stages: “1. Tell us more about you;” “2. About us;” “3. Our results;” and “4. Resources.” First, information about the participants was obtained, after which information about the researcher who conducted the study was presented. Next, text and figures directly from the study, as well as a model of the potential impact of sodium reduction or fiber increase in a fictitious product on the public’s consumption, were presented [40]. This model was a fictional estimate of how a change in the nutrient content and thus FOP information of a bread product, adjusted for sales and consumption, would be reflected in changes in sodium and fiber intake. A figure illustrating their product’s ranking in terms of fiber and sodium content compared to competitors’ products was also presented. The platform ended with potential concrete solutions to implement nutrient changes (e.g., alternative ingredients) and an awareness that businesses could have a significant impact on public health, even by implementing minor changes. A certificate of participation was granted upon completion via the platform.

In addition, a semi-structured interview guide was developed (Appendix A) based on the four stages of the platform [41]. Fifteen questions were available in any of the four stages of the platform, in addition to eight specific questions for Stage 1, three questions for Stage 2, 19 questions for Stage 3, and nine questions for Stage 4. Before the first interview, a pilot test conducted with two professionals working with food companies in an innovation support service affiliated with the research center confirmed that the online platform and interview guide were easy to understand.

To obtain an optimal diversity of participating businesses, the sampling process was conducted in two phases with two sets of criteria for recruitment. For both recruitment waves, the CTAQ supported discussions between the research team and CBQ businesses with the CTAQ proposing that CBQ members participate in the research project. A document explaining the mandate, deliverables, and criteria was presented. The names of interested members were supplied by the CTAQ to the research team, who then contacted the members by telephone to introduce the project and assess their eligibility. If CBQ members were willing to participate, they were sent a consent form, and a date was set for the interview with the research team. All interviews were set to last 90 min in total. Overall, 10 participants were recruited and included in the final analysis, representing nine food businesses, and all participants agreed to participate. With this final sample size, saturation of the major themes was achieved when analyzing the data, so no additional recruitment took place [42].

The first set of participants (*n* = 6) was recruited via convenience sampling [43] through contact with the CTAQ in October and November 2019. To be eligible for this study, all participants were required to be working at a food business that either produced bread or provided ingredients for bread products sold in Canada. To help meet this criterion, the participants had to be members of the CBQ. Within the companies, individuals needed to work in one of the following sectors, which were considered complementary and relevant to food product improvement: management, R&D, marketing/sales, operations, production, or product evaluation. The individual interviews were face-to-face and took place in the participant’s office during their business hours, in Québec, Canada.

Following this first sample and analysis of the data collected, a second set of participants (*n* = 4) was recruited using theoretical sampling [43] through the extended network of the CTAQ in July 2020. The aim of this second set of participants was to add greater sample diversity regarding participants’ roles in businesses and business size, as the businesses recruited thus far did not vary sufficiently in terms of these two criteria. Participants were classified as either belonging to either a large business (500 employees or more) or a small-to-medium enterprise (SME) (between 10 and 499 employees) [44], to guide the recruitment of this second set of participants. The second set of interviews was conducted during office hours, but through video conference due to COVID-19 restrictions.

During the interviews conducted by researchers M.L.B. (registered dietitian and graduate student), J.L. (registered dietitian research professional) and M.T. (registered dietitian research professional), the participants were asked to think aloud as they experienced the online platform [38]. This method provided insights into how they processed the information (content and format) and their reasoning while exploring the platform. In addition to probing participants about what they were saying aloud, the interviewer selected questions to generate discussions about the platform in general, nutrition improvement, and technical aspects of the website based on the interview guide, following the best practices for semi-structured interviews [41]. Although the questions may have varied from participant to participant, all themes relevant to the analysis were discussed.

Audio recordings of the interviews were transcribed verbatim by a contracted research assistant with guidelines based on the method put forward by Bazeley [45], and participants were assigned numbers to protect their confidentiality. All transcripts were checked by interviewers for completeness and accuracy prior to data analysis but were not sent to participants for validation. For the analysis, researchers were first guided by a general inductive approach [46]. This allowed the participants to have a strong voice in articulating the results of this study, as this method allows new ideas to emerge from a specific context, as opposed to affirming existing concepts in the literature. Throughout the data analysis, researchers were careful to maintain their reflexivity through reflective writing, the use of a journal to capture thoughts during coding for each coder, and collaboration within the research team to discuss key findings, as they were close to the team that had conducted the original study presented on the platform, and had to minimize the impact of their biases, personal experience, and prior knowledge of the situation [47, 48].

The researchers used thematic content analysis to code the data, in which themes were coded as a unit of analysis after which their frequency was considered [49, 50], as described in other qualitative studies [12, 51, 52]. All interviews were first open-coded by two researchers using NVivo10 (QSR International). They individually coded each verbatim transcript, merged their analyses and discussed discrepancies to reach agreement for each code in each interview. They subsequently developed second-order themes as first-order themes became saturated, and additional interviews were coded. Only themes reported by seven or more participants are presented in the results section to focus on the most salient themes and impart sufficient depth and detail to convey the richness and complexity of our data, while avoiding “thin” codes [53]. Participants were treated as an homogenous group, as their characteristics were useful for recruitment and diversifying the sample in terms of roles in the organization and business size for the theoretical sampling but were not used to create sub-groups. Attempts to group respondents into smaller groups created clusters that were too small or had no relevant differences. Finally, through discussion within the research team, a structure emerged from the themes as a conceptual model. Participants did not provide feedback on the findings.

## Results

**Fig. 1:**
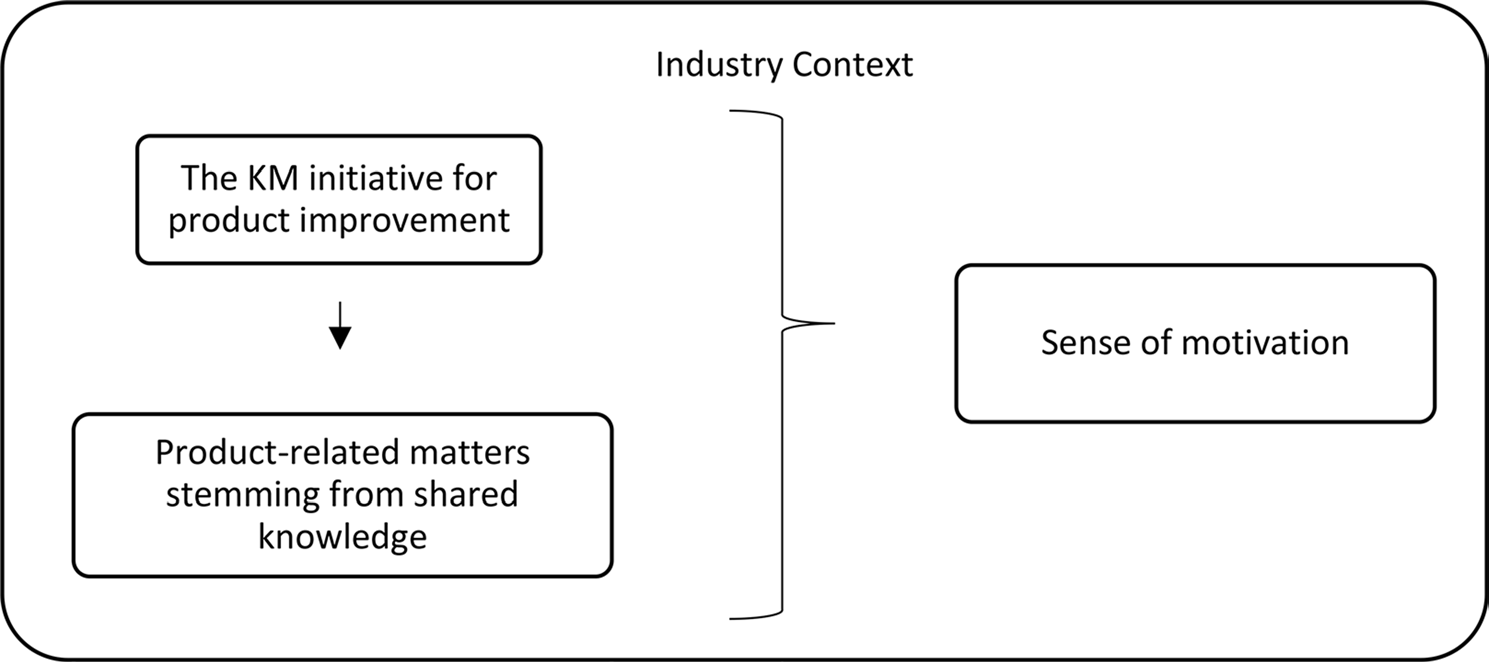
Schematization of the four final salient themes emerging from the analysis

Table 1 first describes each participant’s key characteristics. Results are then presented according to the final themes in the resulting conceptual model, namely, the industry’s context, the KM initiative for product improvement, the product-related matters stemming from shared knowledge, and the sense of motivation. Tables 2, 3 and 4, and 5 present more details on each of these themes, descriptions and sample quotes. Finally, the resulting conceptual model is presented in Fig. 1. All the original quotes are in French and were translated into English by the researchers.

### Participant characteristics

Of the ten participants who were interviewed, five were from ingredient supplier businesses and the other five were from producer/supplier businesses. Seven participants worked in marketing/sales, two in R&D, and one in management. Of the nine different food businesses represented (two participants were from the same business), four were SMEs and five were large businesses. In terms of organizational hierarchy, seven participants worked in the management of a sector or in general management, two were heads of a sector, and one was an employee. Seven had between five and twenty years of experience in their sector, while the other three had fewer than five years of experience. (Table 1).

**Table 1:** Key participant characteristics (*n* = 10)

Participants	Business type	Sector in the business	Business size	Hierarchy in the Business	Years of experience
1	Ingredient supplier	Marketing/Sales	Large business	Management of a sector	5–20
2	Producer/Supplier	Marketing/Sales	SME	Management of a sector	5–20
3	Producer/Supplier	Management	SME	General management	5–20
4	Producer/Supplier	Marketing/Sales	SME	Head of a sector	0–5
5	Ingredient supplier	Research and development	Large business	Management of a sector	5–20
6	Ingredient supplier	Research and development	Large business	Employee	0–5
7	Ingredient supplier	Marketing/Sales	SME	Management of a sector	5–20
8	Ingredient supplier	Marketing/Sales	Large business	Management of a sector	5–20
9	Producer/Supplier	Marketing/ Sales	Large business	Head of a sector	0–5
10	Producer/Supplier	Marketing/ Sales	Large business	Management of a sector	5–20

### Industry context

All participants discussed the context of their organization during the interviews (Table 2). For all participants, the importance of customers was central to their decision-making process, even for business-to-business (BtoB) businesses, which have other businesses as customers, or business-to-consumer (BtoC) businesses, which have consumers as customers. Influence of other actors was also considered and discussed as being entirely part of a business’ current context. Notably, Participant #1 mentioned Health Canada as an actor that could influence their context, and that communication was sometimes difficult. They also spoke at length about their reality, including their experiences, competitors, values, and practices. Interestingly, the role of the baker in the development of recipes and its influence on the nutritional quality of products emerged as salient in the business discourse.

**Table 2:** Themes, descriptions and sample quotes associated with the industry context

Themes	Descriptions	Sample quotes
Importance of customers	Excerpts that show the role of consumers relative to industry context. Examples include consumer’ knowledge, their needs and choices, the business’ reputation with consumers, etc.	“Is the consumer willing to pay more for a product that is more interesting nutritionally?” (Participant # 6)
Influence of other actors	Comments on other organizations (political, public, or private) and/or on scientists who influence their context. Examples include Health Canada, MAPAQ, universities, dietitians, etc.	“Well, Health Canada, that’s what’s a bit… [hesitation], it’s always a bit special with Health Canada: they’ll allow, you know, things that you wonder why they allow, and they’ll abolish certain things that you don’t understand why they abolish them, you know, sometimes they don’t have eh… they don’t have an incredible communication (with us), I think.” (Participant # 1)
Their reality	Excerpts that address the subject of industries and their own internal context. Examples include the presence of competitors, personal skills of the individual interviewed, strategies in the business, role of the baker in product improvements, etc.	“I am in marketing and sales (…) maybe it would be relevant, given that we are talking about bread, to approach bakers. To see the relevance with them at the level of the recipe development or at the nutritional level, to know what they think of it. Basically, they are the ones who create the bread. It’s them… It’s thanks to them that the bakeries are there. Without bakers, we don’t have a bakery, and we don’t have a store.” (Participant # 4)

### The KM initiative for product improvement

Participants characterized many sub-themes regarding the knowledge mobilization web platform initiative and how the information had been communicated (Table 3). Most participants would have preferred a more collaborative approach, using a more interactive platform to generate the scientific results presented. For example, Participant 2 felt that he had to accept not only the results, but also the approach. Moreover, the only two nutrients addressed were sodium and fiber, and he questioned the prerequisites of the observational study. Participants commented that the online platform was too long. Regarding the shared information, adapting the message to their reality was also important to keep them interested in the study. For example, it was relevant to have information about the Québec market, as most product data are usually from the US market. In addition, participants emphasized having the information properly explained, such as knowing the nutritional standards and the recommendations on which they are based. Finally, the need for synthetization and clearer objectives was important, such as determining what exact quantity of sodium per slice is considered healthy.

**Table 3:** Themes, descriptions and sample quotes associated with the KM initiative for product improvement

Themes	Descriptions	Sample quotes
Collaborative approach	Excerpts mentioning that the platform is not participatory, collaborative, or interactive enough. Excerpts indicating a need for more collaboration on the platform, as well as regarding the generation of the scientific results presented.	“Well, that’s how I understand it, with so many steps, it’s not an interactive platform in the easy sense of the word, it’s not… it’s not multiple, it’s one, it’s monomaniacal, there’s a way. You have to go in, go through these steps from 1 to 10 or that’s it, it’s academic […] here, I have to accept an approach and the prerequisites that it is fiber and sodium, the two spearheads of the nutritional value, and that bread is not good in sodium, it is not good in fiber. You see, there are many prerequisites.” (Participant #2)
Online platform is too long to consult	Comments on the time it takes to navigate the platform or the length of the text - preferably, it should not be too long.	“Too many words. I have to go see some other videos; I don’t have time to read right now. It’s a big day, I have 12 meetings, I have 8 people coming into my office at the same time they all want the same thing [Sigh].” (Participant #7)
Need for an adapted message and results	Need to rephrase words for more nuance in the results or to have an adapted message to reflect the participants’ reality.	“The Québec market is really a special one. Often, we look at the results for the United States. Even, even honestly in the United States, I don’t understand how they manage to have a result that summarizes the whole country because there are so many different markets that it’s difficult to do so in my opinion. But that’s, that would be a plus, that it’s adapted to the market here, in my opinion.” (Participant #10)
Need to have properly explained information	Need to know what is true from false, to have the truth, the reference, more scientific information, etc.	“I would like to know, okay, but four grams, uh, what is the basis for the percentages of daily value? You know it’s always a bit confusing, uh, the percentages of the daily value don’t tell the whole story either. Personally, I don’t eat 2,000 calories every day, so what does that mean? So, what is it based on? So, a lot of people won’t know that the percentage of the daily value is based on an a… on a 2,000 calorie diet.” (Participants #6)
Need to synthesize and have clear objectives	Mention of a desire for clear goals, limits or thresholds, or concrete actions to be taken and/or to reduce the amount of information.	“Well, the conclusion, nearly half of the sliced breads in our Québec sample contain too much sodium according to the usual health recommendations’’ (reading on the screen) and how much is too much? It’s how much that we aim for because, we need salt in a bread recipe… what’s your average? I’m going to say 2 grams, and then well, in general we find 4 grams. Well, to know we have to reduce by half, that would help. For conclusion two: ‘’Almost ¾ of the sliced bread.‘’ Same thing for conclusion two, it’s a nice conclusion but, how much per slice do you think is good?” (Participant #3)

### Product-related matters stemming from shared knowledge

Many product-related questions emerged from the knowledge shared on the platform, indicating the implications of the shared information for their products (Table 4). First, participants explained that a modification to a nutrient, either fiber or sodium, would modify the shelf life of bread products, as well as its taste and texture. In addition, participants mentioned that there were many labelling and regulatory implications to consider before making any changes; in particular, the effect that a warning symbol (FOP) would have on their package, and how relevant it would be to recognize it. It is also important to consider the vocation of a product. For example, if a product is created to be tasty rather than healthy, changing its purpose to be more nutritious may impact its taste. Finally, three types of needs were mentioned to be able to modify the nutritional quality of their products: financial resources, material resources, and more information, particularly regarding alternative ingredients.

**Table 4:** Themes, descriptions and sample quotes associated with product-related matters stemming from shared knowledge

Themes	Descriptions	Sample quotes
Changes to shelf life	The impact on conservation of the modification or development of a new recipe with less sodium or more fiber.	“I have a very biased position with respect to sodium in bread dough because I know the nightmares [emphasis on the word] that it causes in the bakery. Especially in sliced bread, where conservation is a point that is existential–and that does not come from the sliced bread industry, but from the grocers who ask that the bread has a shelf life of more than 5 days. If we decrease the quantity of salt, it has a direct impact also on conservation.” (Participant #7)
Changes to taste	Mention that reducing sodium or fiber (or any other modification) changes the taste of the bread.	“White bread doesn’t taste like anything if there’s no salt, honestly [laughter]. It’s like eating eh, cucumbers–let’s say, natural, without anything, you add salt so the taste of the cucumber explodes [laughs].” (Participant #1)
Changes to texture	Mention that reducing sodium or fiber (or any other modification) changes the texture of the bread.	“Sodium is essential to the control of my production and the fibers make my starch bellows heavier, but I need salt to densify it, so [by removing salt], I remove the cement from my product matrix, in scientific terms.” (Participant #2)
Labelling and regulatory implications to consider	Anything related to improving food product labelling (Nutrition Facts table, list of ingredients, nutrition claims) and associated regulations.	“I think it might be relevant to show how scary it [a warning symbol about the nutrient content] can be on a package. You know, how you don’t necessarily want that [emphasis added] on your package. So uh, maybe that could be relevant [to add].” (Participant #6)
Consideration of the vocation of a product	Reference to the positioning of the product on the market, it’s vocation (e.g., healthy product).	“We position the health aspect at the top versus pleasure versus gourmet […] for sure if my goal is to respond to people who are price sensitive and therefore to have the least expensive bread possible, well, my priority will certainly not be to have [good] nutritional values.” (Participant #10)
Need for financial resources	Mention of the need for funding to improve or develop new products, e.g., access to scholarships, grants or financial aid, or cheaper ingredients. Clear mention of a need for financial resources.	“We work a lot with external laboratories that we pay, and that we pay a lot of money for. Like this week we sent samples to test the content, basically the quality and the fiber content, uh, fiber, sodium, sugar, fat, and protein, of like 30 products. I think it costs us about $9,000 […] that’s a lot of money. So, if we can somehow get access to some of the analysis, uh, at a reduced cost, that would be great, everybody would win.” (Participant #9)
Need for material resources	Mention of the need for material resources to improve the products. For example: special ingredients, machinery, storage, etc.	“Maybe there is an accessible software that could be purchased [for recipe management]? I think the CTAQ could buy the software, then we would be 25 companies to use it, or we would have a monthly fee to use it.” (Participant #4)
Need for more information	Mention or statement of a need for additional or necessary information on the platform to improve the products. The need must be clearly stated, either explicitly or via a question.	“You could make a hyperlink there, to a database where there are details and advantages/disadvantages of different ingredients suggested to improve the nutritional quality of breads.” (Participant #5)

### Sense of motivation

Throughout the text, excerpts about the participants’ motivation to improve the nutritional quality of their products were coded bimodally, with first-order themes detailing what motivated them (Table 5).

All participants were motivated to improve the nutritional quality of their products. For example, participants felt that it was right that actions were being taken to improve the food supply quality. Two clear motivations stood out: participants felt motivated when they could see an impact on public health and when a change might bring them new clients (data not shown).

Simultaneously, some participants felt less motivated about improving the nutritional quality of their products. There were no specific, clear-cut reasons for being less motivated that surfaced in the analysis; however, many individual reasons were given by the participants. For example, a sense that it might not be the role of their business to improve public health, but that their priority is to make bread to be eaten.

**Table 5:** Themes, descriptions and sample quotes associated with sense of motivation

Themes	Descriptions	Sample quotes
More motivated to improve the nutritional quality of their products	Quotes mentioning that the business is motivated to improve the nutritional quality of their products.	“Given that we know that there are so many health problems related to food and it’s very, very expensive. I think it’s a good investment to have governments that are involved in improving the supply of food on the table and that people are aware of it.” (Participant #8)
Less motivated to improve the nutritional quality of their products	Quotes mentioning that they are not motivated to improve the nutritional quality of their products.	“It’s not that we don’t care about these things, it’s just that, in the end, we make bread to be eaten. That’s our priority. Even beyond making money, the goal is that when you create food, you don’t want to throw it away at the end of the day.” (Participant #6)

### Resulting conceptual model

The resulting conceptual model, presented in Fig. 1, summarizes the four final themes presented and identified as salient in the participants’ discourse during the interviews: the context of the industry’s KM initiative for product improvement, product-related matters stemming from shared knowledge, and sense of motivation. The themes are presented with associations to show how they were conceptualized relative to each other. First, the industry context was pervasive in the participants’ discourse. As a result, this theme is conceptualized in the background and encompasses other themes. The KM initiative itself (i.e., the online platform) was another major theme characterized by participants, as well as considerations of product-related matters stemming from shared knowledge. The first concept relates to how the information was communicated, and the second relates to the implications of that information for the participants. Notably, the arrow is unidirectional from the KM initiative for product improvement to product-related matters stemming from shared knowledge. This is because, without the presence of the KM platform and the information shared, product issues would not have emerged in the conversation. Both themes reported the usefulness of the platform and the information shared. These three themes were related to how participants expressed their sense of motivation for the KM initiative throughout the interviews, as they mentioned that they were either more motivated or less motivated to improve the nutritional quality of their products.

## Discussion

This study provided a portrayal of themes for researchers to consider to better mobilize public health nutrition knowledge with food industries to improve the nutritional quality of products. We presented four salient themes resulting in a conceptual model from the food industry representatives’ discourse: how their industry’s context, the KM initiative itself, and product-related matters stemming from the shared information contributed to their motivation to improve the nutritional quality of their products. For each theme, we presented the theme, description and sample quotes. Our study provides insights into a KM initiative between public health nutrition researchers and the food industry, which has not been done before. It also allows for the comparison of previous general insights from other contexts with this specific one, such as the importance of universities’ knowledge mobilization capabilities and their dynamics in the innovation ecosystem to promote social change [54], or the importance of high consumer demand for product innovation for the success of industry-university collaboration [55]. Furthermore, our study highlights key considerations for when the scientific knowledge shared with food industries is nutrition sciences rather than food sciences, or food engineering knowledge. Regarding the case studied, we also specified key considerations before suggesting any changes in sodium and fiber in bread, as well as technical features to consider when developing an online KM platform for the food industry.

The theme industry context, a topic discussed in other work related to the use of knowledge assets in organizations and in open innovation [56, 57], showed that customers appeared central to the industry’s discourse in improving the nutritional quality of their products, as they are mentioned both in context and as a source of motivation. This also resonates with other studies in which consumer acceptance is paramount before any food reformulation is considered [14, 58–60]. Indeed, sodium reduction, an improvement suggested in the current KM approach, is known to be accepted by consumers [61]. This is consistent with our theme of “product vocation”, which implies that products on the shelf will not change if there are still profitable consumers for them. Thus, as a more healthy and socially conscious generation of consumers enters the market [62], the industry might develop healthier products to meet consumer demands if they see the opportunity for more revenue. Industries could gain a competitive advantage in collaborating with public health researchers, as innovative and healthy product ideas can potentially increase sales [63]. In our data, being unable to sell an improved product is one aspect of not being motivated to improve the nutritional quality of their product. The opposite is also true, where it is a motivator if it can increase sales. Thus, researchers who wish to engage in KM initiatives should be aware of the importance of the consumer to industries and can present consumer data and potential profit-generating opportunities to get their attention and motivate them to improve the nutritional quality of their products.

Regarding the KM initiative itself, the participants in our study mentioned that they would have preferred a more collaborative approach. Our mobilization could be described as end-of-grant knowledge transfer (KT), where we involved knowledge users only when the results were generated, versus an integrated KT, where the knowledge users are involved early in the research design [64]. An integrated KT approach usually fosters research that is more relevant to the knowledge users because they are involved in every step of the research project. This finding is consistent with common guidelines for effective knowledge mobilization, which recommend meeting with knowledge users, especially at the beginning of a project [65]. Nevertheless, regarding food systems in particular, the frequent association of the food industry with researchers has led to fierce criticism of their proximity and influence, as well as the potential to distort scientific results [25, 30]. Good ethical practices should always be implemented to ensure that such collaborations are conducted with high scientific rigor. Cullerton and colleagues found a high level of agreement on the principles relating to standards of research governance, transparency, and publication in the literature on partnerships between public health researchers and food industries [66]. However, there was less agreement on the appropriateness of industry collaboration [66]. As such, Hawkes and Buse suggest involving food industries only after public health objectives have been set [24]. Researchers must be aware that integrating food industries prior to the generation of results can be beneficial to KM itself. However, it must be conducted with due regard to the independence and integrity of the results and be carefully executed.

As for the technical aspects of the KM approach, such as the length of the platform and the need for resources and clearer messaging, synthesized information and adapted messages could easily be improved, according to the participants. For example, working with a marketing/communication agency or science education organization could improve the communication aspect of KM. Researchers could also use the help of the technology transfer office or other similar intermediaries, where they may find useful allies in creating better industry-targeted communication [67, 68]. Researchers should collaborate with other professionals and seek help to develop a well-executed KM initiative online or in person and avoid developing it independently.

In terms of product issues arising from the information shared on the platform, many points were raised from a food science perspective specifically related to shelf life, taste, and texture. Working with food technologists or chefs (e.g., directly with bakers) could help anticipate obstacles that public health researchers might face in suggesting a product improvement that may not be technically feasible. Providing potential solutions could enhance motivation and improve absorptive capacities for new knowledge among industries [10, 13, 69, 70]. Another solution may be to provide companies with resources where they can obtain ideas on how to implement a suggested improvement. The need for resources, whether financial, physical, or informational, was also echoed in previous work on university-industry collaboration, where factors conducive to successful collaboration include the availability of resources to pursue a collaboration [71]. Researchers need to focus on product-related issues and a lack of resources, either by providing additional food science knowledge or sources of help and resources, to improve the likelihood that nutritional knowledge will be used.

Regarding motivation, legislation was mentioned both as a part of their motivation and as a product-related matter. The upcoming front-of-pack (FOP) regulation in Canada, which was launched in June 2022, will identify products that are high in total sugar, saturated fat, and sodium [72], which may be a concern for industries. Our data show that this could entice industries to change their products (i.e., reformation) since they want to avoid this warning on their packaging. Other studies have found results consistent with this effect, where food patterns are improved after the implementation of FOP labelling [73, 74]. This study also highlights key elements for improving public policy, providing insight into sensitive industry issues regarding nutritional improvements. Any regulations regarding FOP labelling could accelerate the pace of collaboration between nutrition researchers and the food industry because of the need for compliance or the fear of losing consumers, who may negatively view products with such labelling.

Finally, our resulting conceptual framework shares similarities with Bacon et al.’s model of conditions for knowledge transfer [75, 76]. The model suggests that a combination of relationship, organizational, and knowledge characteristics contributes to successful knowledge transfer among members of an open innovation ecosystem. In this model, the “learning intent” of organizations [77], i.e., the willingness to learn new knowledge, is crucial for successful knowledge transfer. This theme is similar to our “sense of motivation” theme, as both themes delineate the motivation to be proactive with the shared information. Bacon et al. also emphasized that “tie strength” can play an important role in successful knowledge transfer, suggesting that actions be taken to strengthen exchanges among organizations. Many studies have highlighted that trust and communication are important to efficiently implement evidence [10, 11, 78]. Building a long-term relationship with food industries could be beneficial in improving the success of KM, as it may help produce a more adapted message and reduce ambiguity [10]. Inter-organizational trust is one of the strongest mechanisms for lowering barriers to university-industry collaborations [79]. As Levin has indicated, simply sharing evidence and asking individuals to change is insufficient [78]. According to our study, we believe that the themes that have emerged could have been better addressed prior to the KM initiative if the research team had built an earlier relationship with the food industries that were considering knowledge mobilization. In particular, we could have understood their realities and the influence of other actors, two themes that have revealed themselves to be significant in their context, along with the other themes. In fact, as general advice, we believe that building a long-term relationship with industries can help any KM initiative by providing insights to address the themes found in this study, as well as to build trust.

The limitations of our study include the limited salience of themes regarding motivators or de-motivators (under the theme of “sense of motivation”), as the probing questions asked during the interviews were not focused on producing sufficient data regarding these sub-themes. Rather, the data found were scattered in the interviews. However, future research could place greater focus on these motivators/limitations with specific questions. Our data can only address the relationship between all themes and the industry’s sense of motivation, and not the specification of that sense with various subthemes. In addition, we presented this concept as opposites, meaning either being motivated or not. Future work could refine this conceptualization as two side of a spectrum under the same theme. Other limitations include the small number of respondents and the lack of characterization of participants’ discourses. It would be interesting to know whether the discourse of SMEs or large companies differ, as they exist in different contexts and these differences might appear, for example, in the necessary resources. Unfortunately, our analysis was not sensitive to this filter, given the small number of participants when grouping by characteristics. Finally, the translation of our participant’s quote from French (the language in which the study was conducted) to English for the purpose of this paper may have influenced the accuracy of the reporting of some of our participant’s discourse. The verification of the quotes by a trained translator might have helped to enhance accuracy. Further research is needed on this topic, since other types of food products and a post COVID-19 context might lead to additional or different insights, as participants’ characteristics and contexts may vary.

## Conclusion

Recommendations for public health researchers and practitioners to better mobilize nutrition science for nutrient improvement with the food industry include providing them with more information about consumer interest in targeted nutrients, using an integrated approach to knowledge mobilization that considers ethics and integrity, working with communication professionals to better disseminate scientific information, working with food science experts to address potential technical barriers to proposed nutrient reformulation, and providing resources, such as financial or material, to act on shared information and support food reformulation. Legislation such as the FOP could accelerate the pace of collaboration between researchers and industry. As this is a case study that raises new questions and hypotheses, future research could further elaborate on these findings with additional in-depth interviews and focus groups with similar participants to solidify the findings and deepen the analysis of the themes highlighted. Overall, it appears of relevance for researchers to build collaborative and transparent relationships with food industry to further address the recommendations resulting from this study and be proactive in their solutions to mobilize the scientific knowledge they generate and contribute to population health.

### Electronic supplementary material

Below is the link to the electronic supplementary material.

Supplementary Material 1

## Data Availability

To protect participant’s confidentiality, interview respondents were assured raw data would remain confidential and would not be shared.
