# Supplementary material for: Knowledge mobilization between the food industry and public health nutrition scientists: findings from a case study
Source: BMC Nutr. 2024 Jun 6;10:81. doi: 10.1186/s40795-024-00889-z (PMC11155086; doi:10.1186/s40795-024-00889-z)
Supplement: Supplementary file 1 — Supplementary Material 1 [file 40795_2024_889_MOESM1_ESM.docx]

**COLLABORATIVE APPROACH WITH THE QUEBEC FOOD INDUSTRY - "MOBILIZING FOR ACTION" PROJECT**

**This project has been approved by the Research Ethics Committee of Laval University: Approval number (#2019-225 A-1/11-05-2020)**

**Interview and observation guide**

**EXPLORATION OF THE KNOWLEDGE MOBILIZATION PLATFORM PROTOTYPE**

**Individual meeting with participants**

**Date: 2019 - _____- _____ Time: _____________________ Location: ___________________________**

**Company no: _____________**

**Participating Member No.: ______________________________________**

**Position: ____________________________________**

**Interviewer: __________________________________**

**Before starting the exploration:**

□ Review confidentiality arrangements with the participant.

□ Verify with the participant that he or she still consents to participate and be recorded (audio).

□ Verify that the recorder is functioning properly.

□ Recall the purpose of exploring the web platform

CAUTION: ‘’THAT’’

If the participant says, "I liked that" while pointing, we need to clarify what "that" is for the recording!

□ Start the recording. Open the platform and let the participant start the experiment

(the sections will scroll on the screen). Record comments as you go (verbal and non-verbal) and ask the following questions throughout the experiment:

**General questions (can be asked in any section, to probe the thinking aloud):**

1. How could this be reworded to make it clearer?
2. What do you understand about it?
3. What don't you understand about it?
4. What do you have in mind when you ask this question?
5. Is there anything else you would like to mention?
6. What do you think?
7. What other information would you miss?
8. What would you find more appealing?
9. Where does your company stand on this information?
10. How do you respond to this information?
11. Can you think of any other issues?
12. Can you think of any other barriers?
13. Do you have any suggestions for improvement?
14. What do you think of the presentation?
15. What do you think of the sequence?

**Questions specific to the sections of the platform:**

**Section 1:**

Home Page

1. Who do you think the platform is for?
2. What do you think of the introduction?

Bread industry page

1. Reading this, what are your expectations?
2. What do you think about the choice of nutrients?
3. What do you think about what you will get with this platform?
4. What do you think of the privacy agreement?

About Us page

1. What is your perception of researchers or the academic field?
2. What do you think the ideal relationship between industry and academia would be?
3. What is your perception of public health?
4. What are your expectations of us?
   - What needs could we help you meet?

**Step 2:**

About You:

1. How motivated are you to change the nutritional value of your breads?
2. How do you feel about the need to answer these questions?

Excellent News Page

1. Do you see any other possible motivations for viewing these results?

**Step 3:**

Our Results

1. What do you understand from these findings?
2. What do you think about the wording?
3. What do you think of this bread classification?

Conclusion Page 1

1. What do you think of this conclusion?
2. Had you seen this figure from Health Canada before (5% is a little, 15% is a lot)? What is your understanding of it?
   1. What do you think about a possible warning symbol on packaging?
3. What do you use in your work?
   1. Do you use the Nutrition Facts table in your work?
      - 1. If not, how could you use it?

Conclusion Page 2

1. What do you think about this conclusion?
2. How do you position yourself in relation to these findings?

What is the potential for improving the nutritional value of your sliced breads?

1. Describe what you need to do at this step?

Your position based on your sodium and fiber content compared to the competition

1. What do you understand about the graph?
2. How do you respond to your position on the graph?
3. How could it be improved to be more understandable?
4. What do you think about seeing the supply of bread in Quebec this way?
5. What use could you make of this graph?
6. How do you position yourself in relation to the competition?

7 reasons why you are an important player in improving the food supply in Quebec

1. How would you characterize your motivation to improve the food supply?
2. What are some other reasons besides those listed?

What if all companies made one small change to improve the sodium and fiber content of their sliced breads, what would be the difference

1. What do you understand about this tool?
   1. How might you use the exported PDF?
2. How do you think the industry will respond to these results?
   1. In terms of motivation for change?

**Step 4:**

Resources

1. What are your resources needed to make a change in your sodium and fiber offering specifically?
2. What do you think about these solution paths?
3. Can you think of any other solution paths to improve sodium and fiber?

How to get into action page.

1. What do you think about these resources?
2. What other resources could they be?

Thank you for experiencing the AFFLUENCE platform page.

1. What do you think would be the incentives for using this platform?
2. What do you think would be the barriers to using this platform?
3. What do you think of the overall tone of the platform?
4. What do you think of the platform versus your needs?
   1. If not answered, what would be better for you?
